# Impact of *Schistosoma* sp., Infection on Biological, Feeding, Physiological, Histological, and Genotoxicological Aspects of *Biomphalaria alexandrina* and *Bulinus truncatus* Snails

**DOI:** 10.1007/s11686-023-00760-4

**Published:** 2024-02-01

**Authors:** Hebat-Allah A. Dokmak, Olfat A. Hammam, Amina M. Ibrahim

**Affiliations:** 1 Medical Malacology Laboratory, Theodor Bilharz Research Institute, Corniche El-Nile St., Imbaba, Giza, 12411 Egypt; 2 Pathology Department, Theodor Bilharz Research Institute, Corniche El-Nile St., Imbaba, Giza, 12411 Egypt

**Keywords:** *Biomphalaria alexandrina*, *Bulinus truncatus*, Genotoxic effect, Feeding, *Schistosoma haematobium*, *Schistosoma mansoni*

## Abstract

**Background:**

Trematode infections of the genus Schistosoma can induce physiological and behavioral changes in intermediate snail hosts. This is because the parasite consumes essential resources necessary for the host's survival, prompting hosts to adapt their behavior to maintain some level of fitness before parasite-induced mortality occurs.

**Methods:**

In this study, the reproductive and biochemical parameters of *Biomphalaria alexandrina* and *Bulinus truncatus* were examined during the cercareal shedding stage of infection with *Schistosoma mansoni* and *Schistosoma haematobium*, respectively, compared with controls.

**Results:**

The study revealed an infection rate of 34.7% for *S. mansoni* and 30.4% for *S. haematobium*. In *B. alexandrina* infected with *S. mansoni*, a survival rate of 65.2% was recorded, along with a mean prepatent period of 30.3 ± 1.41 days, a mean shedding duration of 14.2 ± 0.16 days, and a mean lifespan of 44.1 ± 0.24 days. Meanwhile, in *B. truncatus* infected with *S. haematobium*, a survival rate of 56.4% was observed, with a mean prepatent period of 44.3 ± 1.41 days, a mean shedding duration of 22.6 ± 2.7 days, and a mean lifespan of 66.9 ± 1.6 days. Feeding increased in both infected species of snails, while the net reproductive rate (Ro) of the infected snails decreased. Total antioxidant (TAO) and lipid peroxidation activity increased in the two infected snail species during shedding, while Glutathione-*S*-transferase levels decreased. Lipid peroxidase activity and nitrogen oxide levels significantly decreased in infected *B. alexandrina* and increased in infected *Bulinus.* Steroid hormone levels were elevated in infected *Biomphalaria,* whereas they were reduced in infected *Bulinus*. Comet assay parameters showed an increase in the two infected genera after infection compared to control snails, indicating genotoxic damage and histopathological damage was observed.

**Conclusions:**

These findings demonstrate that infection with larva species diverse biochemical, hormonal, genotoxic, and histopathological changes in the tissues responsible for fecundity and reproduction in *B*. *alexandrina* and *B. truncates* comparing with controls.

## Introduction

Schistosomiasis is a chronic parasitic disease caused by trematodes of the genus *Schistosoma*. It is considered the second most devastating disease worldwide in terms of morbidity and mortality [1, 2]. This disease is prevalent in tropical and subtropical areas, affecting approximately 240 million people globally, with about 700 million people at risk, particularly in poor communities with inadequate sanitation facilities [3–6].* Schistosoma mansoni* and *S. haematobium* are the two parasites that cause the most widespread forms of intestinal and urogenital schistosomiasis [7]. In our laboratory, we use the infection of *B. alexandrina* with *S. mansoni* and *B. truncatus* with *S. haematobium* to study the impact of host-parasite infections on physiological and behavioral changes, including reduced fecundity and increased feeding behavior in the two intermediate host species. When *B. alexandrina* becomes infected with *S. mansoni* and *B. truncatus* becomes infected with *S. haematobium*, the development of the hermaphroditic reproductive system of the two snail species is severely retarded [8–10], resulting in the production of almost no eggs. Numerous studies have reported behavioral alterations in hosts, such as changes in feeding and crawling behavior, caused by parasitic infection, and have interpreted these changes as induced adaptations by parasites to facilitate transfer to the next-stage hosts [11–14]. Increased feeding with infection has been interpreted as compensating for nutrient deprivation caused by parasites or as a modification of the host's growth rate (gigantism) [15, 16]. The comet assay has several advantages over other DNA damage methods, such as sister chromatid exchange, alkali elution, and micronucleus assay, due to its high sensitivity and the ability to determine DNA strand breaks in individual cells [17–19]. Gastropod snails have been reported to be intermediate hosts of certain larval digeneans [20, 21]. These snails harbor various developmental stages, such as sporocysts, rediae, and cercariae. During their multiplication and growth, they obtain nutrients from infected tissues, such as the digestive gland and gonads, leading not only to diverse histopathological changes in the snails but also to physiological disturbances [19, 21–24].

The aim of this study was to expand and update the existing knowledge regarding behavioral alterations in hosts, specifically focusing on feeding and fecundity, caused by parasitic species. The *B. alexandrina-S. mansoni* and *B. truncatus-S. haematobium* models were utilized for comparison with uninfected species (controls). In addition, biochemical, histopathological, and genotoxic parameters were measured in the tissue homogenate of both infected and uninfected snails to facilitate the comparison.

## Material and Methods

### Species Snails with Infections

Juvenile specimens of both *B. alexandrina* (shell diameter 3–5 mm) and *B. truncatus* (shell diameter 3–5 mm) were obtained from the stock reared in the Medical Malacology Department at Theodor Bilharz Research Institute (TBRI), Imbaba, Giza, Egypt. The snails were originally collected from field populations in Giza Governorate and were used for all experiments. The snail species were bred under standard conditions according to the methodology described by [25].

To induce infections, triplicate groups of 10 *B. alexandrina* snails were individually exposed to 5–8 freshly hatched *S. mansoni miracidia*, and triplicate groups of 10 *B. truncatus* snails were individually exposed to 8–15 freshly hatched *S. haematobium miracidi*a for 3 h at 25 °C in 2 ml vials containing dechlorinated tap water, following the protocol outlined by [10]. Miracidia of *S. mansoni* and *S. haematobium* were obtained from the Schistosome Biological Supply Center (SBSC) at Theodor Bilharz Research Institute in Egypt. Triplicate groups of 10 control snails were individually placed in 2 ml vials without exposure to miracidia. Both infected and control snails were housed in plastic aquaria (10 snails per container, with a size of 16 × 23 × 9 cm) filled with dechlorinated water. The infected snails were allowed to develop for 4 weeks after infection with *B. alexandrina* and 8 weeks after infection with *B. truncatus.*

The infection rate was calculated 4 weeks after infection in *B. alexandrina* and 8 weeks after infection in *B. truncatus*, following the method described by [26]: Infection rate = (number of infected snails/total number of snails examined) × 100. The survival rate at shedding was also calculated for both snail species according to Frank (1963) using the following equation: survival rate = $\frac{{\text{Number of survived species snails}}}{{\text{total number of exposed miracidia species snails}}} \times 100$. Furthermore, the mean total number of cercariae, mean duration of shedding, mean prepatent period and mean lifespan were calculated for each species with positive infections, following the approach by [26].

In experiments involving quantitative cercarial counting, the standard exposure time was extended to 45–60 min. The water containing cercariae from the test tubes was carefully transferred into Petri dishes lined with graduated paper. To immobilize and stain the cercariae, a few drops of Lugol’s solution (7.5 g KI + 5 g I2 in 100 ml distilled water) were added, facilitating rapid visualization. The cercariae were then counted under a dissecting microscope [27].

### Snails Feeding

Approximately 120 snails of the same size (3–5 mm) from each species were used in the infected and control groups. They were housed in a plastic container (16 × 23 × 9 cm) and provided with 50 circles of washed clean fresh lettuce leaves measuring 4 mm^2^. The snails were starved for one day before the experiment, and then the food was given [28]. The consumption of the lettuce circles was counted and recorded daily, and the number of surviving snails in both species was noted [29]. Triplicate groups were performed for each species and compared side by side with the control groups.

### Biological Parameters

To investigate the fecundity of the two snail species, Styrofoam sheets measuring 5 × 5 cm with a thickness of 0.5 cm were used as substrates for egg deposition. These sheets were placed on the water surface of plastic containers. Weekly collection of egg masses was carried out for a period of four to eight consecutive weeks. The egg-laying capacity was quantified as (Mx), which represents the total number of eggs laid in a given week divided by the initial number of living snails (eggs/snail/week) [30]. The survivorship of the snails (Lx) and the total number of eggs laid per snail (Mx) were recorded on a weekly basis for each aquarium. The net reproductive rate (R0) of the snails throughout the experimental period was calculated using the following parameters: Survivorship (Lx), which represents the proportion of snails that survived at any given week relative to the initial population (1.0 = 100% survival rate), and Fecundity (Mx), which refers to the average number of eggs laid per snail per week. The net reproductive (R0) at any given period was determined using the formula (R0 = ΣLxMx).

## Species Snail Tissue Homogenates and Biochemical Estimations

To investigate changes in biochemical parameters TAO, LPO, SOD, NO, and GST in two infected species of snails, three replicates of 10 snails per liter were prepared for two infected species at the cercarial shedding stage, as well as two control groups of the tested species. Snails with an average shell diameter of 7–8 mm were carefully crushed between two glass slides, and their shells were removed. Tissue weighing 0.1 g from each species was then homogenized in 1 ml of phosphate buffer (pH 7.1), followed by centrifugation at 4000 rpm for 15 min. The resulting supernatant was collected in Eppendorf tubes and stored at − 20 °C for further analysis.

For the biochemical analyses, Biodiagnostic kits (Biodiagnostic Dokki, Giza, Egypt) were employed to determine the levels of SOD and GST [31, 32]. Tissue malondialdehyde (lipid peroxide) was assessed according to the method described by [33]. Nitric oxide (NO) concentration was determined using a colorimetric NO kit (Biodiagnostic Company, Dokki, Giza, Egypt; Cat. No. GR 2511), based on the approach outlined by [34]. Additionally, the total antioxidant capacity was estimated using a kit (Cat. No. TA 2513) following the methodology established by [35].

### Steroid Sex Hormones (Testosterone and 17β-Estradiol)

The study aimed to assess the levels of steroid hormones, specifically testosterone and 17β-estradiol, in the tissues of two snail species: one infected with the trematode species and another serving as an uninfected control group. The hormone levels were measured using the T EIA kit from Enzo Life Science (Michigan, USA, ADI-900-065) and the E EIA kit from Cayman Chemical Company (Michigan, USA, item no. 582251) according to the instructions provided by the manufacturers [36].

### Genotoxicity by Comet Assay

A study was conducted to compare DNA damage in snails infected with trematodes at the shedding stage as well as a control species group, following the methods described by [37, 38].

### Histopathological Alterations

The experiment included simultaneous positive infections of *B. alexandrina* with *S. mansoni* and *B. truncatus* with *S. haematobium*, along with their respective control groups. Each group consisted of three replicates with 10 snails per liter. To examine the snails' tissues, the digestive and hermaphrodite glands were dissected from their shells, fixed using Bouin’s fixative, and embedded in wax blocks. Sections of 5–8 µm thickness were prepared and stained with haematoxylin and eosin, following the protocol by [39]. Similar preparations were made for the control snails' digestive and hermaphrodite glands.

### Statistical Analysis

The values of biological and biochemical parameters were expressed as mean ± SD (standard deviation). Statistical analysis was performed using the student's "*t*" test to determine significant changes between the control and infected groups, following the method by [40]. The limit for statistical significance was set at *p* < 0.05, corresponding to a confidence level of 95%.

## Results

### Snail’s Infection Rate

**Fig. 1:**
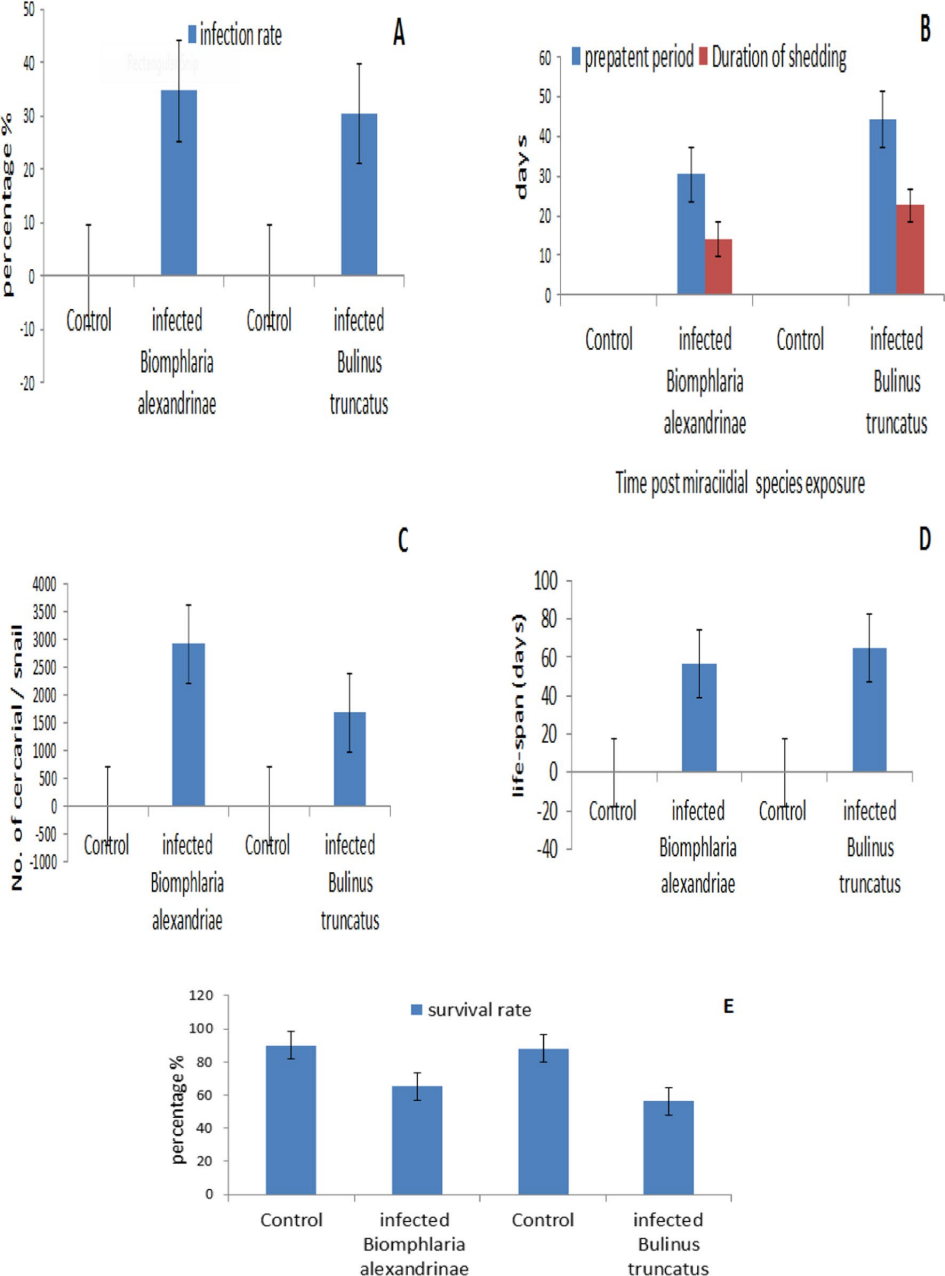
Impact of *S. mansoni* with *B. alexandrina* and *S. haematobium* with *B. truncatus* on infection rate (**A**), pre-patent period and duration of shedding (**B**), total cercarial production (**C**), life span post miracidia species exposure (**D**) and Snail’s survival rate 1st cercarial shedding stage (**E**) comparing with uninfected snails.

The infection rate in *B. alexandrina* with *S. mansoni* was recorded as 34.7% (Fig. 1A), while *B. truncatus* with S. haematobium had a recorded rate of 30.4%.

### Prepatent Period and Duration of Cercarial Shedding in Snails

The pre-patent period varied from 28 to 32 days (mean: 30.3 ± 1.41) post-infection for *B. alexandrina*, and it was recorded as 43–50 days (mean: 44.3 ± 1.41) post-infection *for B. truncatus* (Fig. 1B) at 25 °C. The duration of shedding ranged from 11 to 29 days (mean: 14.2 ± 0.16) in *B. alexandrina* and from 14 to 36 days (mean: 24.6 ± 2.6) in *B. truncatus.*

### Mean Total Number of Cercariae Per Snail

The mean number of cercariae per snail (Fig. 1C) in *B. alexandrina* was recorded as 2915 ± 74.394 (*p* < 0.05), and it was recorded as 1637.3 ± 307.5) (*p* < 0.05) in positive *B. truncatus*. Cercariae production typically increased after the first week of patency but often decreased significantly towards the end of the snails'.

### Snail’s Mean Life Span

The mean lifespan was recorded as 44.1 ± 0.24 days in *B. alexandrina* (Fig. 1D) and 66.9 ± 1.6 days in *B. truncatus*.

### Snail’s Survival Rate at First Shedding

The survival rate of *B. alexandrina* exposed to *Schistosoma mansoni* at the first cercarial shedding was 65.2%, while the survival rate of *B. truncatus* exposed to *S. haematobium* was 56.4%, compared to the survival rate in the respective control groups (Fig. 1E).

### Impact of *Schistosoma mansoni *with *Biomphlaria alexandrina *and *S haematobium *with *Bulinus truncatus* on Feeding, Fecundity and Reproductive Rate

**Fig. 2:**
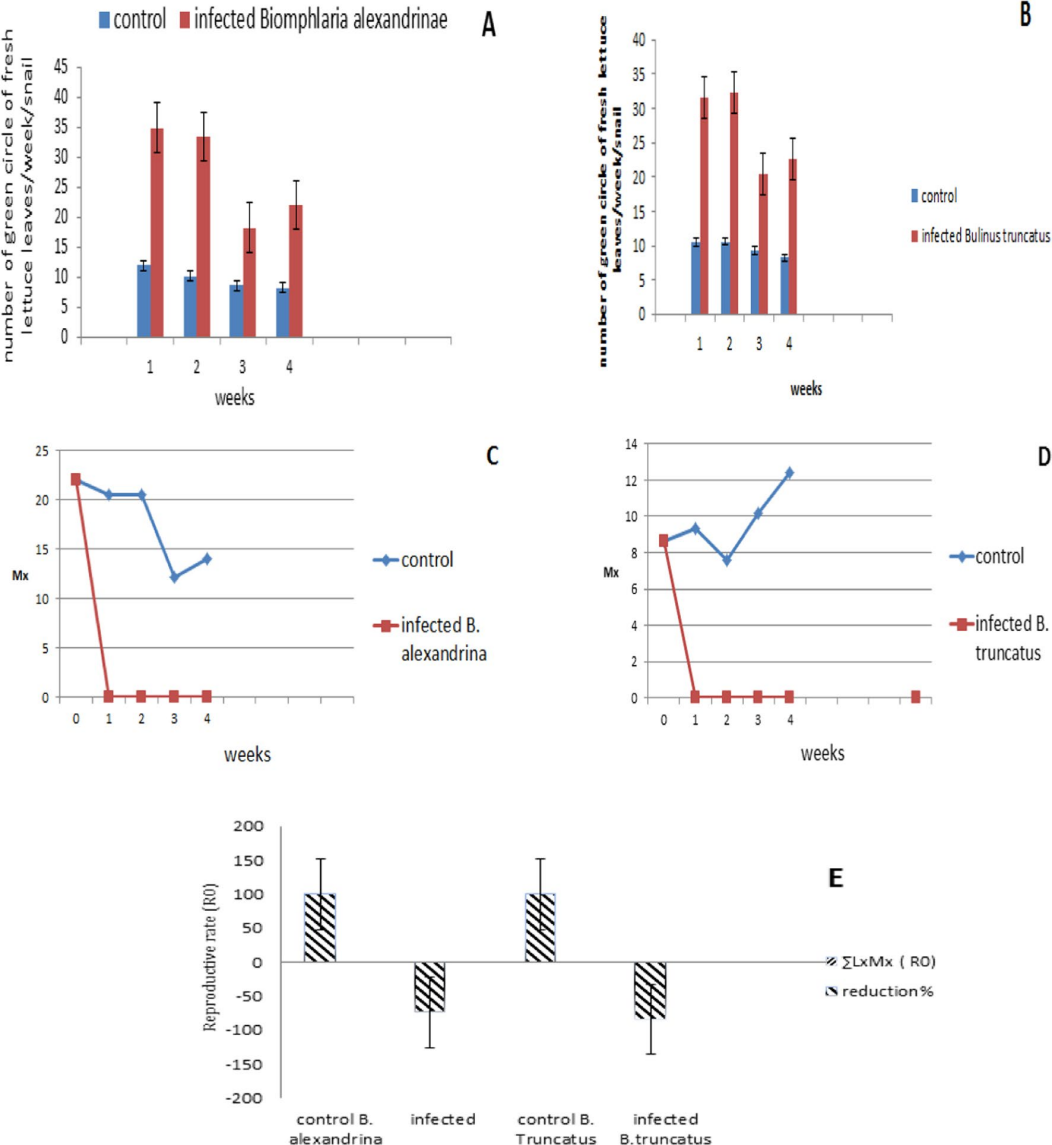
Impact of *S. mansoni* with *B. alexandrina* and *S. haematobium* with *B. truncatus* on feeding (**A**, **B**); on fecundity (**C**, **D**) and reproductive rate (**E**) in two infected snails, Survivorship (Lx): This represents the proportion of snails that survived at any given week relative to the initial population (1.0 = 100% survival rate). Fecundity (Mx): this refers to the average number of eggs laid per snail per week. The net reproductive rate (R0) at any given period was determined using the formula (R0 = ΣLxMx) comparing with uninfected snails

During the prepatent period, the number of feeding *B. alexandrina* snails on green circles of fresh lettuce leaves exceeded that of their uninfected counterparts, indicating that the infected snails were more voracious feeders (Fig. 2A). The same pattern was observed in *B. truncatus* infected with *S. haematobium* (Fig. 2B). Additionally, the fecundity of *B. alexandrina* showed a pattern of ceasing egg-laying for 4 weeks during the prepatent period (Fig. 2C), which was also observed in *B. truncatus* after being exposed to miracidia (Fig. 2D). The net reproductive rate (*R*_o_) in infected *B. alexandrina* and *B. truncatus* was significantly reduced to 47.7% and 84.6% of its value in the respective control groups (Fig. 2E).

## Impact of Infection with *Schistosoma mansoni* in *Biomphlaria alexandrina* and Infection with *S haematobium* in *Bulinus truncatus* on Oxidative Stress Parameters at 1st Cercarial Shedding Stage

**Fig. 3:**
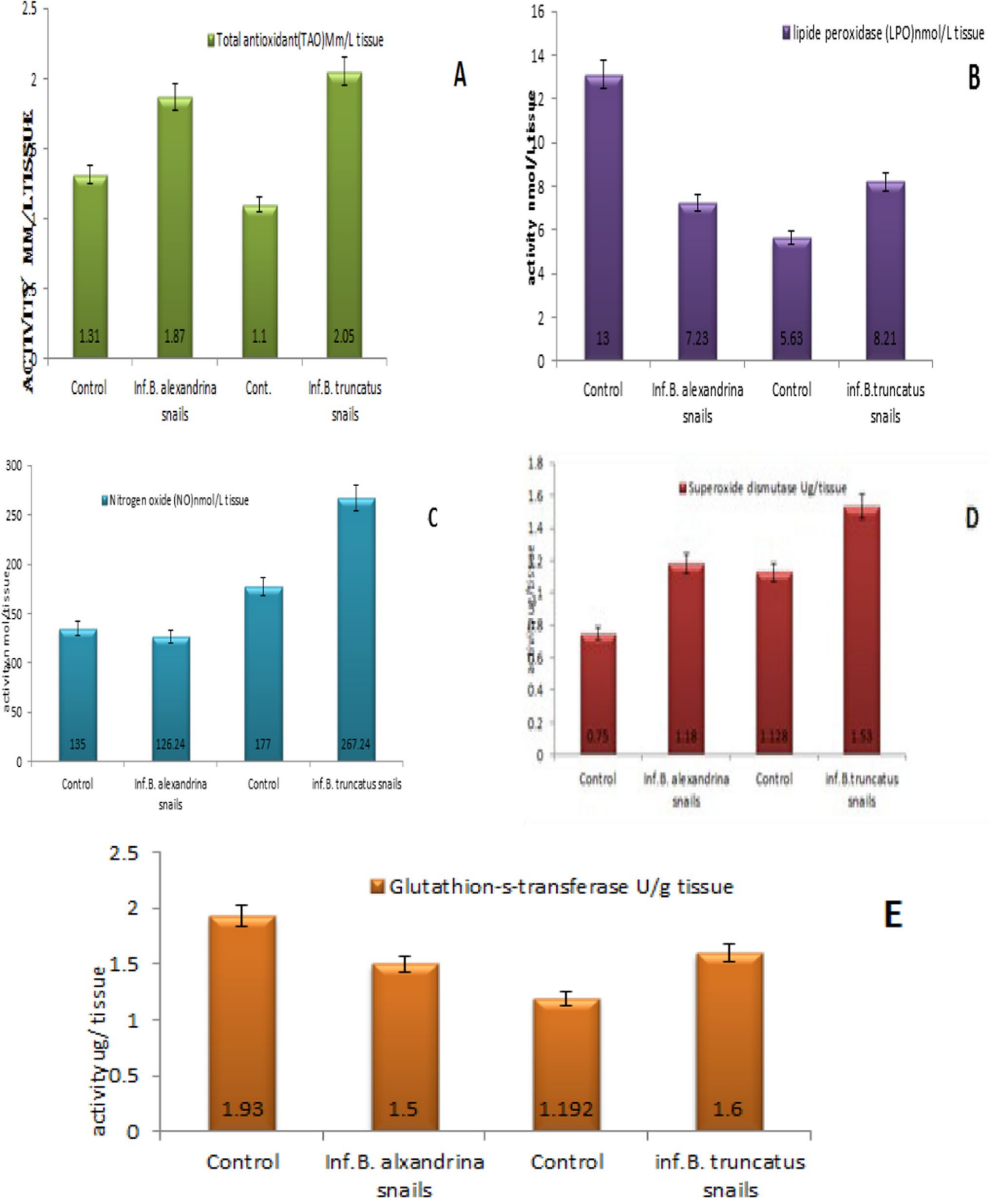
Impact of *S. mansoni* with *B. alexandrina* and *S. haematobium* with *B. truncatus* on total antioxidant (**A**), lipid peroxidase (**B**), nitrogen oxide (**C**), superoxide dismutase (**D**) and glutathione-*s*-transferase (**E**) at 1st cercarial shedding stage comparing with uninfected snails

TAO activity showed a significantly higher value (*p* < 0.05) in the homogenized tissue of *B. alexandrina* compared to the uninfected group. Similarly, infected *B. truncatus* snails exhibited a significantly higher TAO activity (Table 1, Fig. 3A). These findings suggest that the infections were stressful for the snails, triggering an increase in their antioxidant defense mechanism.

**Table 1:** Impact of infection with *Schistosoma mansoni* in *Biomphlaria alexandrina* and infection with *S haematobium* in *Bulinus truncatus* on oxidative stress parameters at 1st cercarial shedding stage comparing with uninfected snails

Intermediate host	Total antioxidant (TAO) Mm/L tissue	Lipide peroxidase (LPO) nmol/L tissue	Nitrogen oxide (NO) nmol/L tissue	Superoxie dismutase (SOD)U/g tissue	Glutathione-*S*-transferase (GST)U/g tissue
Cont. *B. alexandrina*	1.31 ± 0.11	13.11 ± 0.009	132.74 ± 0.004	0.75 ± 0.001	1.93 ± 0.001
Inf. *B. alexandrina*	1.87 ± 0.12	7.23± 0.005	126.24 ± 0.005	1.18 ± 0.001	1.79 ± 0.001
Cont. *B. truncatus*	1.10 ± 0.005	5.63 ± 0.004	176.74 ± 0.005	1.12 ± 0.002	1.19 ± 0.004
Inf. *B. truncates*	2.05 ± 0.009	8.21 ± 0.007	267.24 ± 0.004	1.15 ± 0.001	1.22 ± 0.0009

Lipid peroxidation (LPO) activity displayed contrasting results between the two snail species. In infected *B. alexandrina* snails, LPO activity was significantly reduced relative to the uninfected group. Conversely, in infected *B. truncatus* snails, LPO activity increased significantly compared to the control group (Fig. 3B). These observations indicate that *S. mansoni* infection in *B. alexandrina* may have a protective effect against lipid peroxidation, while *S. haematobium* infection in *B. truncatus* may induce oxidative damage. Furthermore, a significant elevation in the levels of nitric oxide (NO) was observed in the tissue homogenate of infected *B. truncatus* snails whereas infected *B. alexandrina* snails exhibited a significant reduction in NO compared to the uninfected group (Fig. 3C).

Superoxide dismutase (SOD) levels were higher in *infected B. alexandrina* and *B. truncatus* snails compared to uninfected snails in both species (Table 2, Fig. 3D). This indicates an up regulation of the SOD antioxidant enzyme as a response to the infections in both snail species.

**Table 2:** Impact of infection with *Schistosoma mansoni* in *Biomphlaria alexandrina* and infection with *S haematobium* in *Bulinus truncatus* on steroid sex hormones in tissues at 1st cercarial shedding stage comparing with uninfected snails

Intermediate host	17β-stradiol pg/ml	Testosterone ng/ml
Cont. *B. alexandrina*	10.256 ± 0.033	10.68 ± 0.235
Inf. *B. alexandrina*	28.154 ± 0.021	30.18 ± 0.086
Cont. *B. truncates*	60.284 ± 0.056	35.1 ± 0.018
Inf. *B. truncates*	15.136 ± 0.009	10.26 ± 0.092

In terms of glutathione-*s*-transferase (GST) activity, the highest value was measured in infected *B. truncatus* snails, while infected *B. alexandrina* snails exhibited a reduction in GST activity compared to uninfected *B. alexandrina* snails (Fig. 3E). These differences in the antioxidant system response may be attributed to variations in laboratory-infected snail species or the longer prepatent period in *B. truncatus* compared to *B. alexandrina*, regardless of the parasite species.

### Impact of *S. mansoni *with *B. alexandrina *and *S. haematobium *with *B. truncatus* on 17β-Esteradiol and Testosterone Hormones in Tissues at 1st Cercarial Shedding Stage

**Fig. 4:**
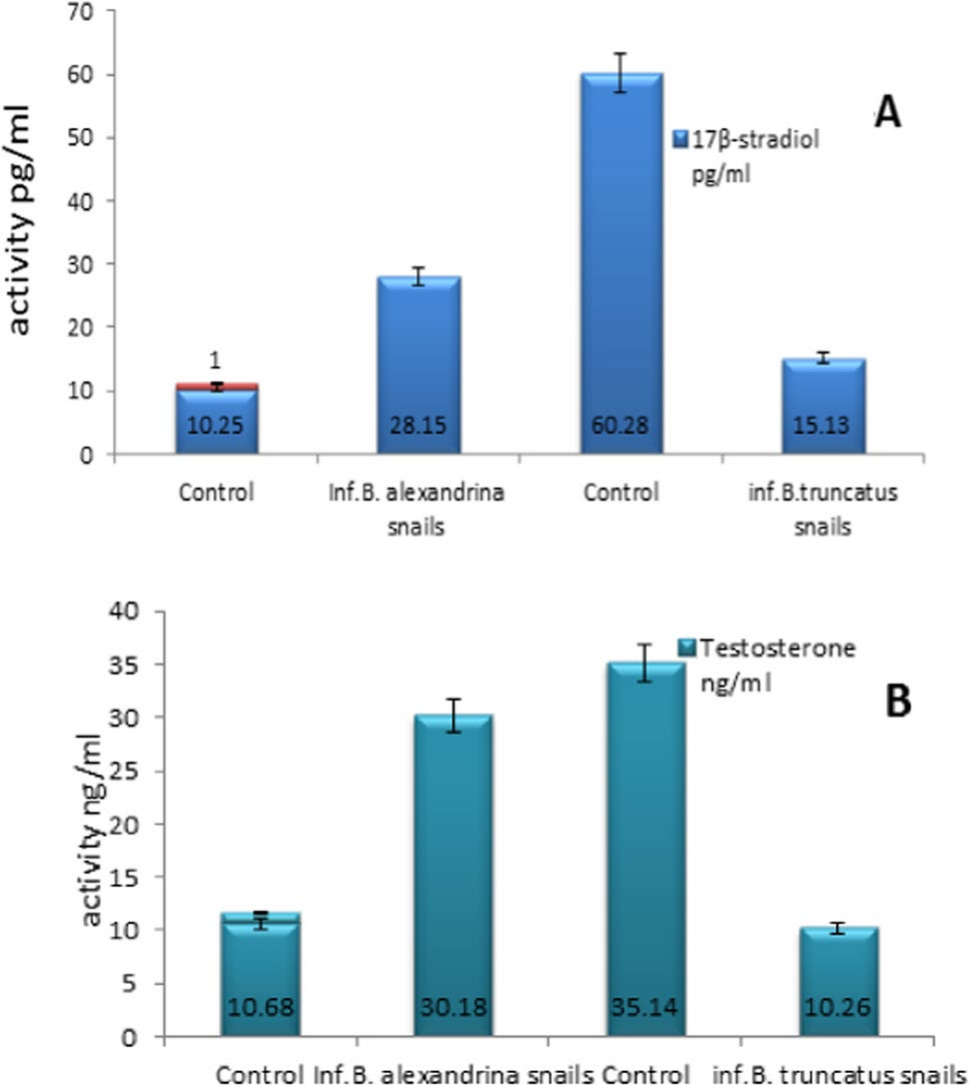
Impact of *S. mansoni* with *B. alexandrina* and *S. haematobium* with *B. truncatus* on 17β-esteradiol (**A**) and testosterone (**B**) in tissues at 1st cercarial shedding stage comparing with uninfected snails

In infected *B. alexandrina* snails, there were significant increases in the concentrations of 17β-estradiol and testosterone in homogenized tissues post-infection (Table 2, Fig. 4A). On the other hand, infected *B. truncatus* snails exhibited a notable reduction in the concentrations of 17β-estradiol and testosterone hormones post-infection compared to their levels in non-infected control snails (*p* < 0.05) (Fig. 4B).

## Impact of *S. mansoni *with *B*. *alexandrina *and *S. haematobium *with *B. truncatus* on Comet Assay at 1st Cercarial Shedding Stage

**Fig. 5:**
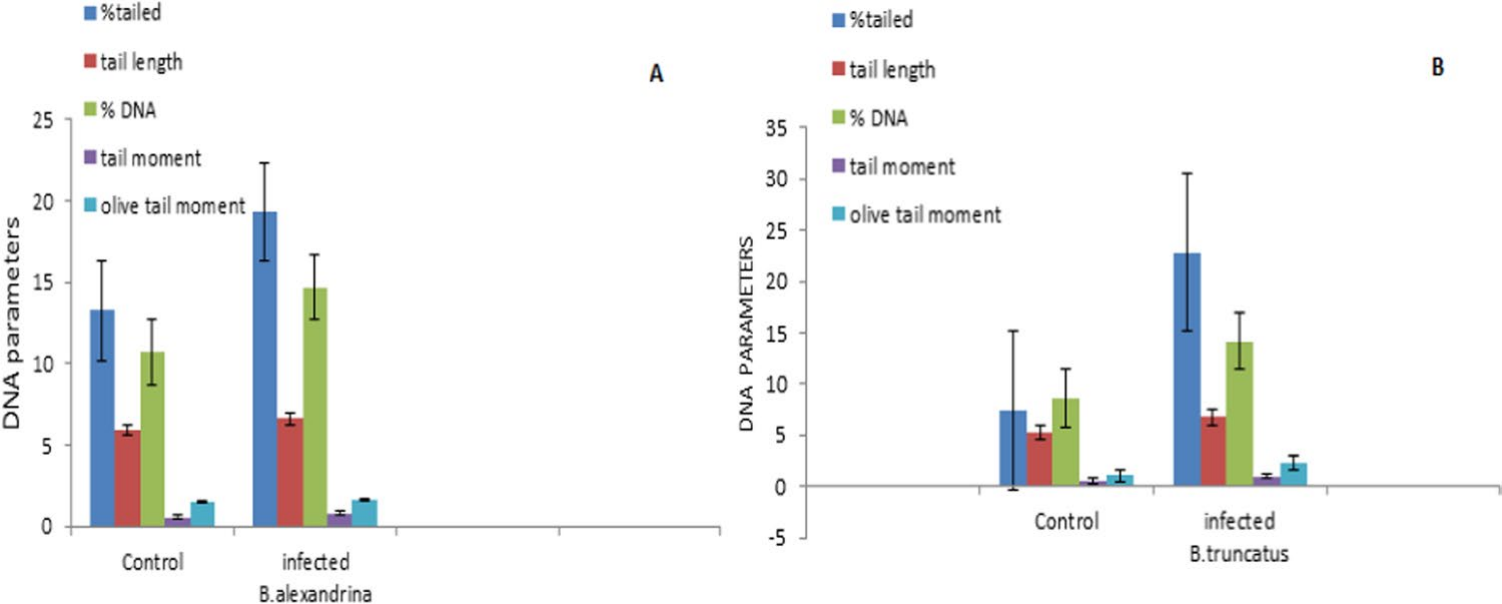
Impact of *S. mansoni* with *B. alexandrina* (**A**) and *S. haematobium* with *B. truncatus* (**B**) on comet assay parameters at 1st cercarial shedding stage comparing with uninfected snails.

**Plate 1:**
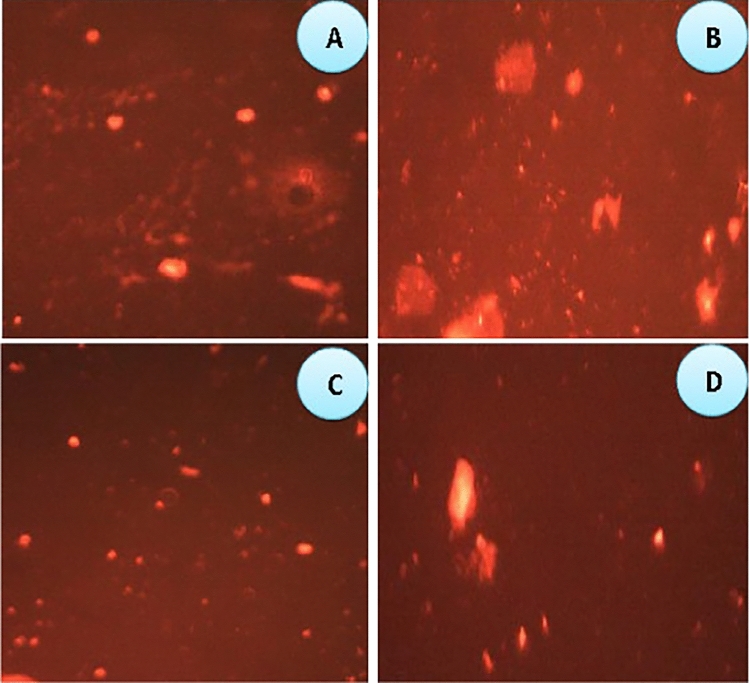
Impact of *S*. *mansoni* with *B. alexandrina* and *S*. *haematobium* with *B. truncatus* on comet assay parameters **A** control* B. alexandrina*; **B** *B. alexandrina*-infected; **C** control* B*. *truncatus *and **D** infected *B*. *truncatus* at 1st cercarial shedding stage

The comet assay was employed to assess DNA damage in *B. alexandrina* and *B. truncatus* snails infected with *Schistosoma mansoni* and *S. haematobium*, respectively. The parameters of tailed % and tailed length, which indicate cellular malformation, exhibited significant increases in infected *B. alexandrina s*nails compared to the uninfected groups (refer to Fig. 5 and Plate 1). Additionally, there was an increase in the percentage of normal DNA in the tail, indicating migration from the head in infected *B. alexandrina* snails. The tail moment, which represents the combination of tail length and the percentage of DNA migrated from the head, may serve as an indicator of genotoxicity and negative effects on the cellular resistance system (Fig. 5 and Plate 1). Moreover, the olive tail moment, a marker of DNA fragmentation, showed a significant increase in infected *B. alexandrina* snails compared to the control group (*p* < 0.05). Similar adverse impacts on comet assay parameters were observed in infected *B. truncatus* snails with *S. haematobium* during the shedding stage (Table 3, Fig. 5A, B, Plate 1).

**Table 3:** Impact of *S. mansoni* with *B*. *alexandrina* and *S. haematobium* with *B. truncatus* on comet assay parameters (DNA) at 1st cercarial shedding stage comparing with uninfected snails

Intermediate host	Tailed %	Tail length(PX)	DNA% in tail	Tail moment	Olive tail moment
Cont. *B. alexandrina*	13.23 ± 0.42	5.9 ± 0.98	10.70 ± 1.54	0.53 ± 0.15	1.47 ± 0.21
Infected *B. alexandrina*	19.3 ± 0.245	6.6 ± 2.032	14.69 ± 0.47	0.79 ± 0.18	1.60 ± 0.13
Cont. *B. truncates*	7.4 ± 0.43	5.22 ± 0.30	8.53 ± 1.77	0.45 ± 0.008	1.07 ± 0.25
Infected *B. truncates*	22.8 ± 0.21	6.73 ± 1.09	14.18 ± 1.16	1.01 ± 0.18	2.30 ± 0.13

## Impact of *S. mansoni *with *B*. *alexandrina *and *S. haematobium *with *B. truncatus* on the Snails’ Digestive and Hermaphrodite Glands Histology at 1st Cercarial Shedding Stage

**Plate 2:**
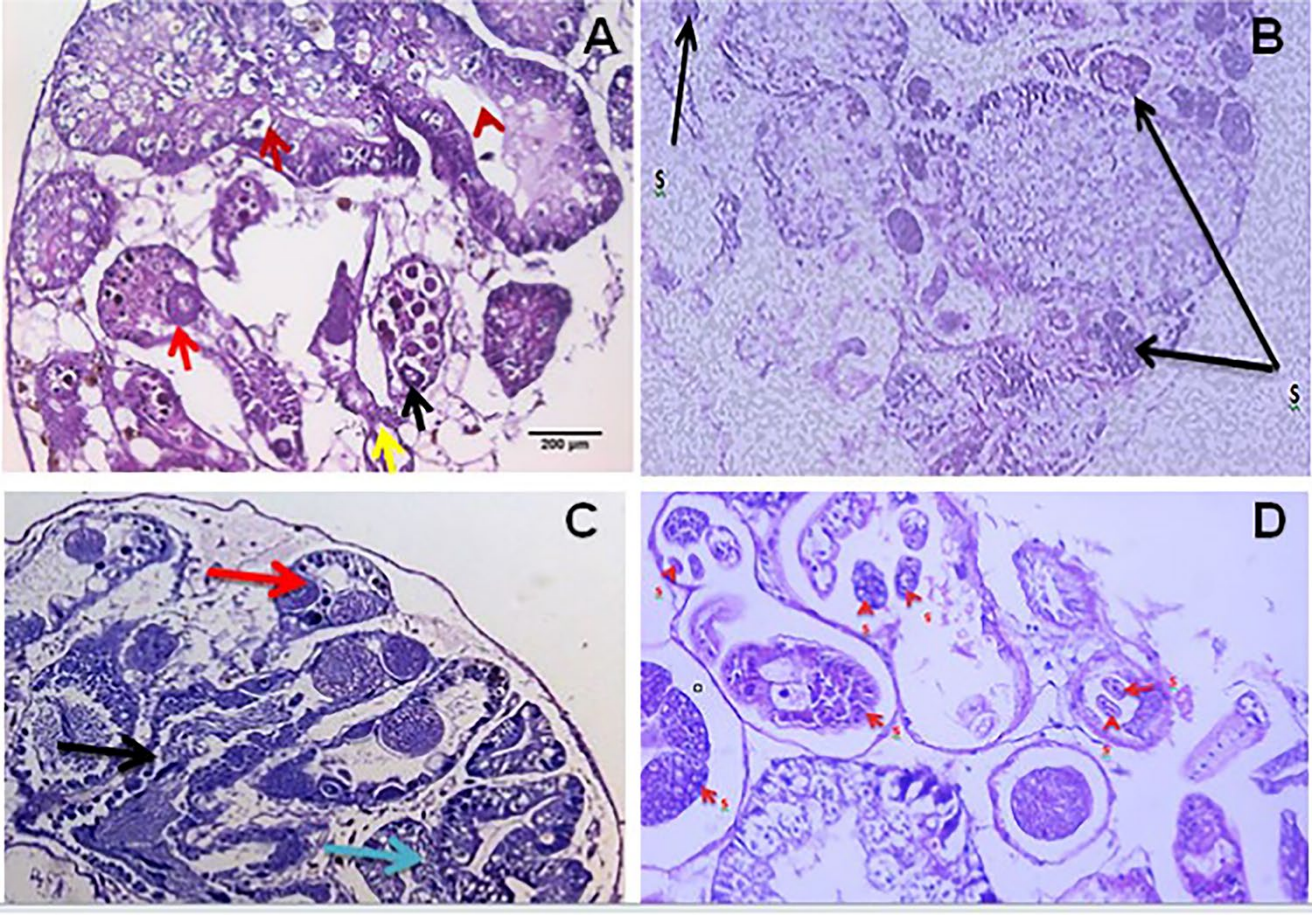
**A** Light micrographs show the normal digestive glands and normal hermaphrodite gland of *B. alexanderina* and **C** normal digestive glands and normal hermaphrodite gland of *B. truncatus* snails. Digestive cells (blue arrow), secretory cells (dark red arrow), Lumen (head dark red arrow) (H&E; × 100; × 200). Mature ovum (red arrow), Oocytes (black arrow), Sperms (yellow arrow). **B** and **D** show infected digestive and hermaphrodite gland where red arrow (s) sporocysts of cercariae species at 1st cercarial shedding stage compared with uninfected snails.

Infection of *B. alexandrina* and *B. truncatus* snails with *S. mansoni* and *S. haematobium,* respectively, can have destructive effects on the snail tissues. Histological studies were conducted on sections from the digestive and hermaphrodite glands of both infected and uninfected snails. The normal histological structure of the digestive gland in both species includes two main cell types: the columnar digestive cells with rounded apices and the pyramidal-shaped secretory cells (Plate 2A & C).

Histological examination of the sections from the digestive gland of infected snails at the shedding stage revealed detrimental effects, including swelling and deformation of the secretory cells, rupturing and disintegration of the digestive cells, as well as the presence of sporecysts containing cercariae species (Plate 2, B & D).

In the hermaphrodite gland, responsible for producing both male and female reproductive gametes, mature ova are located at the periphery of the acinus, while bundles of male sperm are arranged in the center. Various stages of sperm and ova development can be observed simultaneously (Plate 2, A & C). Histological sections of this gland from infected snails showed varying degrees of degeneration in ova and sperm, depending on the sporecysts of cercariae species during the experimental shedding period in both species (Plate 2, B & D).

## Discussion

### Snail’s Infection Rate

Lab observations of *B. alexandrina* infected with *S. mansoni* and *B. truncatus* infected with *S. haematobium* were consistent with the findings of [41], which reported a 30% infection rate (IR) in *B. pfeifferi* snails with *S. mansoni*. However, the observations differed from those of [42, 43], who reported higher IR in *B. pfeifferi* snails infected with *S. mansoni*. In the case of *B. truncatus*, a 50.5% IR was observed in snails aged one to seven days, and a 19.9% IR was observed in snails aged one and a half to 5 weeks under laboratory conditions [44].

### Prepatent Period and Duration of Cercarial Shedding in Snails

The mean pre-patent and Snail’s duration periods for positive *B. alexandrina* and *B. truncatus* observed in this study are consistent with previous findings regarding the time interval between miracidial infection of the intermediate host and the subsequent release of cercariae. Previous research by [45] reported that *S. mansoni* exhibits the fastest rate of development, taking approximately 33 days at 25 °C, while *S. haematobium* takes around 50 days. These findings highlight the significance of prepatency periods in the epidemiology of schistosomiasis, as acknowledged by [46].

Furthermore, [45] emphasized the crucial role of the latent period (prepatent period) in determining the prevalence of infection within snails. The latent period refers to the time interval between snail infection by a miracidium (the larval stage of a parasitic trematode) and the initiation of cercarial shedding (the subsequent larval stage that is infective to the final host). The mean total number of cercariae per snail observed in this study differs from the findings reported by [47] in *B. glabrata*.

However, it aligns with previous studies on *Bulinus truncatus* infected with miracidia, which reported a range of 29–65 days for cercarial production at 24–26 °C [27, 48]. In the present study, the number of cercariae shed weekly by positive *B. alexandrina* was greater than the number of cercariae shed weekly in positive *Bulinus truncatus*. This difference can be attributed to the varying doses of miracidia given to the two species. Massoud [49] demonstrated that the numbers of cercariae shed daily by single snails exposed to one or two miracidia were significantly lower than those exposed to 5, 10, or 20 miracidia.

### Mean Total Number of Cercariae Per Positive Snails

The examination of cercariae in the present study revealed that 90–100% of mature *S. mansoni* and *S. haematobium* cercariae were shed within 45–60 min of exposure to light. Pflüger [27] documented that the standard stimulation period for mature *S. haematobium* cercariae was limited to 5 h. While cercariae production typically increased after the first week of patency, it often decreased significantly towards the end of the snails' lifespan.

### Snail’s Mean Life Span Comparing with Uninfected Snails

The lifespan of Schistosoma-positive snails in *B. alexandrina* was found to range from 45 to 81 days (with a mean of 44.1 ± 0.24 days), while in *B. truncatus*, it ranged from 55 to 91 days (with a mean of 65.9 ± 1.6 days) compared to uninfected snails in both species. The differences between the mean lifespan of infected snails and non-infected snails in the control group in both species were statistically significant (*p* < 0.05). Chu et al. [50] demonstrated that the cercaria-shedding period and the lifespan of infected snails were shorter than those of the non-infected controls.

The longer lifespan observed in *B. truncatus* may be attributed to the higher doses of *S. haematobium* miracidia compared to *S. mansoni* miracidia within *B. alexandrina*. Notably, [51] reported observations on the development of the parasite in relation to tissue changes and mortality among infected snails. It was concluded that the extensive migration of large numbers of cercariae, along with the intense tissue reactions associated with trapped and degenerating cercariae, are significant factors contributing to the death of the snails. Furthermore, laboratory studies conducted by [50, 52] clearly demonstrate that infection with any of the three principal species of human schistosomes adversely affects the survival of the molluscan host.

### Snail’s Survival Rate at at 1st Cercarial Shedding Stage Comparing with Uninfected Snails

This study reported a decrease in the survival rate of two snail species after shedding cercariae, which supports the findings of [53]. The aforementioned study observed lower survival rates in snails exposed to *S. mansoni* miracidia compared to unexposed snails. Previous laboratory studies have shown a wide range of mortality rate increases in schistosoma-infected snails compared to uninfected ones, with some estimates reaching up to 0.100 [45]. Additionally, [54] discovered that patent infections of *S.* species led to higher per capita mortality rates in *Bulinus globosus* and *B. pfeifferia*, including mortalities during the prepatent period in the two infected species. The reduction in this biological parameter may be attributed to potential competition between the parasites and the host for essential haemolymph-borne nutrients [55]. Additionally, it could be a result of histopathogenic effects on the snail host and depletion of nutrients by the parasite, particularly around the time of infection maturation and cercariae shedding [56].

### Impact of Schistosoma Infection on Feeding, Fecundity and Reproductive Rate Comparing with Uninfected Snails

Infected *B. alexandrina* and *B. truncatus* snails exhibited a tendency to feed more frequently compared to uninfected snails. This finding aligns with [57], who observed that freshwater snails infected with larval trematodes displayed increased feeding behavior during the light period under laboratory conditions. Parasite infection often leads to alterations in host behavior, indicating adaptive manipulation of the host behavior by the parasite to enhance its transmission success [58–60].

Increased feeding behavior in infected individuals has been interpreted as a compensatory response to nutrient deprivation caused by parasites or as a modification of the host's growth rate, such as gigantism [15, 16]. Other researchers have described the reduced fecundity in infected snails as castration, suggesting that the trematode parasite alleviates the energetic demands of reproduction, allowing the host to allocate this energy towards other life-history traits, such as growth and survival [61, 62]. Another possible explanation for increased feeding is starvation autolysis, which occurs due to the compression of digestive tubules at various locations, hindering the passage of food into the tubules. This can lead to intracellular digestion, and heavy infection can result in the atrophy of digestive tubules [63, 64]. Infection with *S. mansoni* or *S. haematobium* miracidia has been observed to cause *B. alexandrina* and *B. truncatus* snails to cease egg-laying after exposure, resulting in a reduction in reproduction [8, 10, 15].

The development of the hermaphrodite reproductive system in *L. stagnalis* infected with *T. ocellata* was severely hindered, resulting in a near absence of egg production [8, 10, 15, 65]. Reductions in fecundity were also observed in three *Bulinus* species infected with S. *haematobium* [66]. The decrease in egg-laying could be attributed to nutrient deprivation caused by the parasite or the dual burden of producing both eggs and parasites, which is not borne by the snail [9, 67–69].

In our present study, infected snails ceased egg-laying in the early weeks of infection, leading to a significant reduction in the average number of eggs per snail in both species. This finding aligns with [69], who attributed the suppression of egg-laying to the indirect effect of trematode larvae on oogenesis, potentially caused by nutrient withdrawal by the parasite or the burden of producing eggs and parasites [67, 69]. Nutrient deprivation may be responsible for the decline in egg-laying, coinciding with the development of sporocysts in the digestive gland [70].

Even a small number of mother sporocysts present during the infection stage could be sufficient to disrupt reproductive processes in the two species. Finally, it should be noted that the molluscan host experiences partial or complete castration following infection [71].

### Impact of Schistosoma Infection on Oxidative Stress Parameters at 1st Cercarial Shedding Stage Comparing with Uninfected Snails

Increasing the level of TAO in infected *B. alexandrina* and *B. truncatus* snails may explain the increase in the number of haemocytes and the generation of large volumes of ROS for defensive purposes to damage or kill the parasite's larvae [72–75].

Gornowicz et al. [76] found significant differences in TAS between control and *P. elegans*-infected *Lymnaea stagnalis* during the initial period of the experiments. TAS was influenced by infection with trematodes in *Biomphlaria galabrata* with *S. mansoni* [77].

*Biomphalaria alexandrina* snails infected with *Schistosoma mansoni* showed a significant reduction in the levels of lipid peroxidation (LPO) and nitric oxide (NO) compared to uninfected snails. This reduction may be attributed to the developing schistosome larvae scavenging nutrients from the snail's hemolymph, resulting in a decrease in the amount of nutrients circulating to the nervous system [78].

Furthermore, another study [79] reported a significant decrease in catalase (CAT) and glutathione (GSH) levels, along with an increase in malondialdehyde (MDA) levels, in the tissues and hemolymph of *B. alexandrina* following infection with *S. mansoni*. However, *B. truncatus* infected with *Schistosoma haematobium* exhibited a significant increase in the levels of LPO and NO compared to uninfected snails at the shedding stage. In another investigation [80], it was observed that *B. alexandrina* snails infected with *S. mansoni* and *B. truncatus* snails infected with *S. haematobium* showed a significant elevation in the activities of glutathione reductase (GR), catalase, and superoxide dismutase (SOD). Changes in the infected snail tissue homogenates were also reported [81]. Upon treatment with sodium fluoride, these altered biochemical parameters were restored to their values in control uninfected snails, indicating the ability of sodium fluoride to inhibit oxidative stress and apoptosis in Schistosoma-infected snails [81]. In response to parasitic infection, both *B. alexandrina* and *B. truncatus* snails increase the activity of their defensive haemocytes, which generate significant amounts of reactive oxygen species (ROS) to damage or kill parasite larvae.

### Impact of Schistosoma infection on 17β-Esteradiol and Testosterone Hormones in Tissues at 1st Cercarial Shedding Stage Comparing with Uninfected Snails

Steroid hormones, such as testosterone and estradiol, were found to be elevated in *Biomphalaria* snails during the shedding stage. According to [82], serum estradiol levels in male mice susceptible to *Taenia crassiceps* (TC) infection increased to levels 200 times higher than their normal values. The authors suggested that the parasite affects the immunoendocrine mechanism, creating a highly permissive environment for its rapid growth. In *Biomphalaria alexandrina*, larval trematode infection disrupts normal reproductive activity. This may explain why *S mansoni* snails increase the activity of steroid hormones, creating a highly permissive environment in ova and sperm, resulting in adverse effects on their physiological activities and defense mechanisms. Consequently, infected snails may cease laying eggs. However, infected positive *Bulinus* snails, the hormones were suppressed. De Jong-Brink [83] illustrated that Schistosomin, a peptide produced by the nervous system of infected snails following schistosome infection, interferes with the host's neuroendocrine system, inhibiting the action of reproductive hormones. Steroid hormones have been documented in various molluscs, including *B. alexandrina* [84–87] and *B. truncates* [88].

Hormonal reductions observed in *Bulinus truncatus* and increases in *Biomphalaria alexandrina* may contribute to fecundity loss in these infected snails [89]. Steroid hormones play an important role in gonad development in snails [90]. Hormone administration, including testosterone, estradiol, and progesterone, has been shown to stimulate spermatogenesis and oogenesis in various molluscan species [91–94].

### Impact of Schistosoma Infection on Comet Assay at 1st Cercarial Shedding Stage Comparing with Uninfected Snails

The study revealed that *B. alexandrina* and *B. truncatus* infected with positive cercariae at the 1st cercarial shedding stage exhibited a statistically significant increase in DNA fragmentation and migration in molluscan tissues compared to the control group. These findings are consistent with previous studies that have reported an increase in tail length (length of DNA migration) in the digestive gland cells of infected snails due to larval trematode infections. Furthermore, the percentage of apoptosis was significantly elevated (58.80%) in the snails infected with larval trematodes compared to uninfected snails (39.59%). The DNA damage and increased apoptosis in the digestive glands of infected snails may result in a decrease in 5-HT (serotonin) and DA (dopamine) concentrations in all tissues throughout the course of infection [80]. DNA has long been recognized as a primary target of age-related cellular damage, and its damage can potentially contribute to the aging process [97]. Additionally, DNA damage has been observed in the hemocytes of *Biomphalaria alexandrina* [95] and *Bulinus truncatus* [96]. In response to parasitic infection, both *B. alexandrina* and *B. truncatus* snails increase the activity of their defensive hemocytes, which generate significant amounts of reactive oxygen species (ROS) to damage or kill parasite larvae. These ROS can potentially be toxic to DNA, leading to DNA oxidation and/or strand breaks.

### Impact of Schistosoma Infection on Digestive and Hermaphrodite Glands at 1st Cercarial Shedding Stage Comparing with Uninfected Snails

The study revealed severe damage to the cell constituents of the digestive and hermaphrodite glands in infected *B. alexandrina* and *B. truncatus* snails caused by trematode larvae. Changes in the digestive glands and ovotestis induced by larval digenean trematode parasites have been reported to depend on the severity of infection, larvae size, and types of larvae [98]. Possible explanations for these alterations include mechanical damage resulting from the migration, feeding, growth, and multiplication of trematode larvae, as well as physiological changes such as autolysis and/or necrosis. Previous studies have shown that redial stages cause more mechanical and physiological damage compared to sporocysts [64, 99].

Rediae engulf the host's digestive cells and utilize hydrolases for extracellular digestion, contributing to physiological damage [100]. It can be assumed that spore larval species observed within the two host cell constituents' tissues in the digestive and hermaphrodite glands are more destructive for the two hosts. Parasitic secretions and excretory products that produce toxic effects may also be contributory factors [101, 102].
